# P-1912. Characteristics and Outcomes of Matched Vaccinated and Unvaccinated Patients Hospitalized for Covid-19 in 27 Hospitals During Brazil's Delta and Omicon Waves

**DOI:** 10.1093/ofid/ofae631.2073

**Published:** 2025-01-29

**Authors:** Eduardo V Moraes, Magda Carvalho Pires, Amanda A A Costa, Unaí Tupinambás, Milena S Marcolino

**Affiliations:** Universidade Federal de Minas Gerais, Belo Horizonte, Minas Gerais, Brazil; Federal University of Minas Gerais, Belo Horizonte, Minas Gerais, Brazil; Medical College of Universidade Federal de Minas Gerais, Belo Horizonte, Minas Gerais, Brazil; Universidade Federal de Minas Gerais - UFMG, Belo Horizonte, Minas Gerais, Brazil; Medical School, Universidade Federal de Minas Gerais, Belo Horizonte, Minas Gerais, Brazil

## Abstract

**Background:**

Brazil faced significant challenges during the COVID-19 pandemic, emerging as one of the most affected countries globally in the number of cases and deaths. In January 2021, the country began the national vaccination campaign against COVID-19. Currently, about 80.2% of Brazilians over six months of age are fully vaccinated.

Few population-based studies have compared clinical characteristics and outcomes among patients hospitalized for COVID-19 concerning vaccination status. This knowledge gap was even greater when an attempt was made to match vaccinated versus unvaccinated individuals to control for confounders. Therefore, this study aimed to compare the clinical characteristics and outcomes of matched vaccinated and unvaccinated COVID-19 in hospital patients.

Flowchart of COVID-19 patients included in the study.

**Figure:**
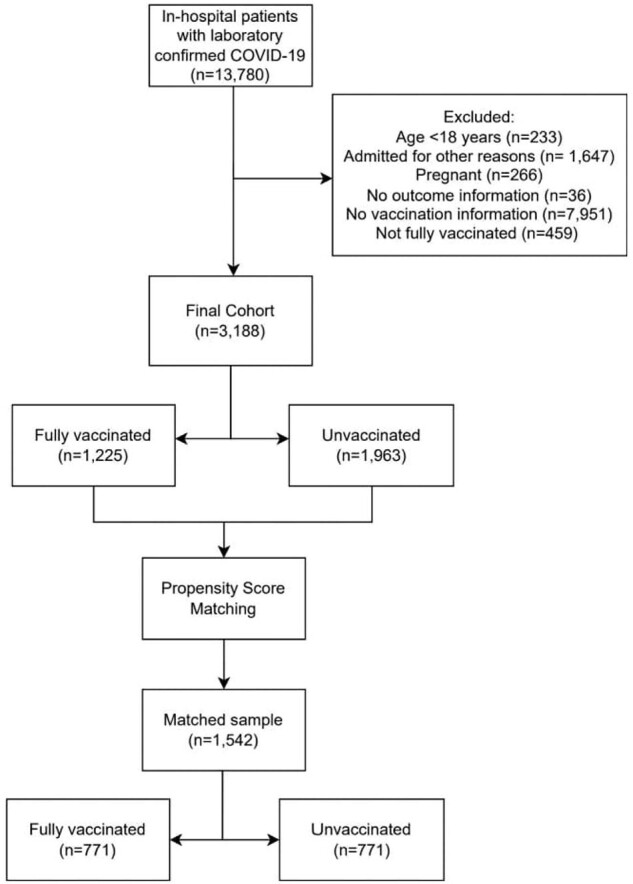

**Methods:**

This retrospective multicenter cohort study included adult COVID-19 patients, with a laboratory-confirmed diagnosis, from 27 hospitals, admitted from March/2021 to August/2022. Clinical characteristics, vaccination status, and outcomes were collected from medical records. Vaccinated and unvaccinated patients were compared in a matched analysis, using the propensity score model that included age, sex, hospital, and comorbidities (hypertension, diabetes mellitus, obesity [body mass index > 30kg/m^2^], heart failure, and chronic kidney disease).

**Figure:**
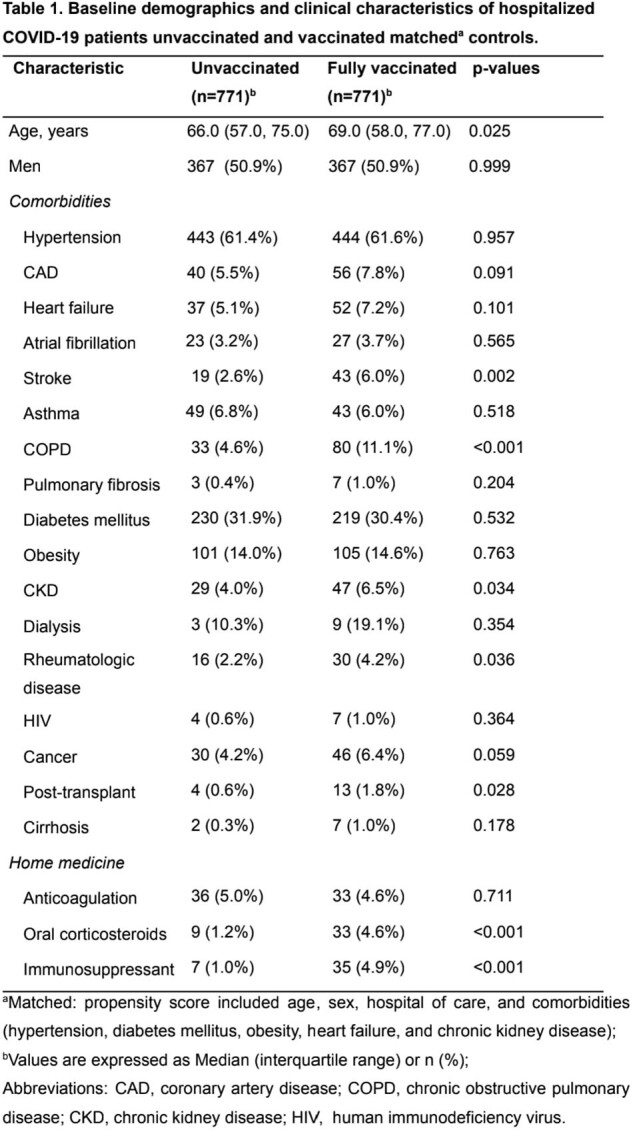

**Results:**

From 3,188 patients, 1,963 (61.6%) were unvaccinated and 1,225 (38.4%) fully vaccinated. Among these, we matched 771 vaccinated with 771 unvaccinated. Vaccinated had lower rates of mortality (17.6% vs. 29.7%), invasive mechanical ventilation (IMV-17.5% vs. 33.0%), non-invasive mechanical ventilation (NIMV- 10.0% vs. 20.2%), intensive care unit admission (ICU- 29.8% vs. 44.0%) vasoactive drugs use (20.5% vs. 30.9%), dialysis (8.0% vs. 13.7%), p < 0.001 for all, and thrombosis (3.7% vs. 7.5% p=0.002).

**Figure:**
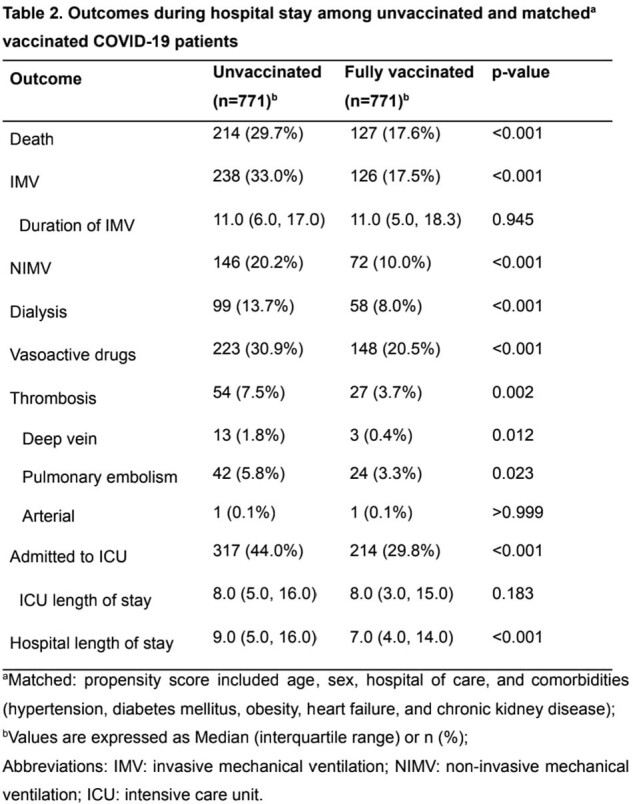

**Conclusion:**

This study suggests considerable benefits of vaccination for patients hospitalized with COVID-19. Fully vaccinated patients experienced lower mortality rates and lower severity disease with fewer ICU admissions, and shorter hospital lengths of stay. These findings confirm that prior vaccination reduces the risk of death and alters the disease trajectory in hospital settings.

**Disclosures:**

All Authors: No reported disclosures

